# Molecular characterization of *Argulus bengalensis *and *Argulus siamensis *(Crustacea: Argulidae) infecting the cultured carps in West Bengal, India using 18S rRNA gene sequences

**Published:** 2016-09

**Authors:** Avijit Patra, Anjan Mondal, Sayani Banerjee, Harresh Adikesavalu, Siddhartha Narayan Joardar, Thangapalam Jawahar Abraham

**Affiliations:** 1 Department of Aquatic Animal Health, Faculty of Fishery Sciences, West Bengal University of Animal and Fishery Sciences, Chakgaria, Kolkata, West Bengal, India; 2 Department of Veterinary Microbiology, Faculty of Veterinary and Animal Sciences, West Bengal University of Animal and Fishery Sciences, Belgachia, Kolkata, West Bengal, India

**Keywords:** Aquaculture, *Argulus* spp, 18S rRNA, Phylogenetic relationship

## Abstract

The present study characterized *Argulus *spp. infecting the cultured carps using 18S rRNA gene sequences, estimated the genetic similarity among *Argulus *spp. and established their phylogenetic relationship. Of the 320 fish samples screened, 34 fish (10.6%) had *Argulus *infection. The parasitic frequency index (PFI) was observed to be high (20%) in *Hypophthalmichthys molitrix *and *Labeo bata*. The frequency of infection was high in September (PFI: 17%) and October (PFI: 12.9%). The 18S rRNA sequences of five *A. bengalensis *(KF583878*, *KF192316, KM016968, KM016969, and KM016970) and one *A. siamensis *(KF583879) of this study showed genetic heterogeneity and exhibited 77-99% homology among the 18S rRNA gene sequences of *Argulus *spp. of NCBI GenBank database. Among the Indian *Argulus *spp. the sequence homology was 87–100%. Evolutionary pair-wise distances between Indian *Argulus *spp. and other *Argulus *spp. ranged from 0 to 20.20%. In the phylogenetic tree, all the crustaceans were clustered together as a separate clade with two distinct lineages. The lineage-1 comprised exclusive of Branchiura (*Argulus *spp.). All *Argulus bengalensis *clustered together and *A. siamensis *(KF583879) was closely related to *Argulus *sp. JN558648. The results of the present study provided baseline data for future work on population structure analysis of Indian *Argulus *species.

## INTRODUCTION

Various infectious and non-infectious diseases are reportedly affecting the cultured fish species, resulting in heavy economic loss to global aquaculture industry [1, 2]. The outer surfaces of the fish are exploited by a huge diversity of parasites including protozoans, monogeneans, leeches, molluscs, crustaceans, etc. The genus *Argulus *(fish louse) of the family Argulidae (fish lice) is one of the most damaging ectoparasites of fish and is of major economic concern in all phases of the aquaculture industry from production to marketing [3]. *Argulus *spp. are the cosmopolitan fish parasites commonly known as fish lice and are distributed throughout the world except Antarctica [3]. These parasites may survive on the freshwater and marine fish species as hosts for most part of their life cycle. It can also survive in variable range of temperatures and its intensity is higher in culture conditions than in natural environment [2-4]. About 129 species of *Argulus *with a complex life cycle involving several metamorphic stages have been reported [3]. *Argulus *spp. cause skin lesions on the host by their suckers and proboscis while feeding, which often lead to secondary infections by bacteria and fungi. Epidemics of argulosis have been reported in carp and trout by producing severe damage to fish stocks [2]. Argulosis cause weight loss by reducing appetite and anaemia in fish. Acute infection of *Argulus *spp. results in fish loss due to direct effects such as dermal ulceration, osmotic imbalance, physiological stress, and immunosuppression [5]. In India, the total loss due to argulosis in carp culture has been estimated to the magnitude of Rs. 29,524.40 /ha/year (1 USD = Rs. 68) [6].

Identification and characterization of *Argulus *has assumed prime importance so that suitable preventive and control measures could be taken up to reduce fish mortality and production loss. Various fingerprinting methods can be broadly classified and genome based approaches are now in use to identify parasites. Genetic data and phylo- geographical structure of a species provide the basic information required to understand their evolution and biogeographical history. The direct sequencing of rRNA gene is generally accepted as a stable and specific marker for identification [7]. Classification of parasites through this technique may be an effectively useful for phylogenetic identification at various levels. Studies on the genetic diversity of *Argulus *spp. and phylogenetic relationship are very limited [8, 9]. A comparative phylogenetic analysis between *Pentastomida *cytochrome C oxidase subunit1 (pcox1), cox2 and NADH dehydrogenase subunits (pnad4) of *A. americanus *was reported [10]. Recently, the genetic diversity of *Argulus *spp. from major aquaculture zones in India by RAPD analysis has been described [11]. Data pertaining to molecular characterization and phylogenetic relationship of Indian *Argulus *spp. are, however, not available. The present study was, therefore, designed to characterize the *Argulus *spp. infecting the cultured carps of India by molecular tools, to estimate genetic similarity within and among *Argulus *populations and to establish phylogenetic relationship.

## MATERIALS AND METHODS

**Sample collection and microscopy: **Samples of live cultured carps, viz., *Catla catla *(n=43)*, Labeo rohita *(n=136)*, Cirrhinus mrigala *(n=41), *Cyprinus carpio *(n=20), *Hypophthalmichthys molitrix *(n=20) and *Labeo bata *(n=60) were collected randomly between June 2012 and March 2013 from Haripota (Lat. 22°31’08”N; Long. 88°28’14’’E), Garia and Chakgaria (Lat. 22°27’55”N; Long. 88°24’24’’E) and Bantala (Lat. 22°31’36”N; Long. 88°26’45’’E) of South 24 Parganas District and Memari (Lat. 23°10’32”N; Long. 88°06’24’’E) of Burdwan District, West Bengal, India (Table 1) for determining the prevalence of *Argulus *infection. A total of 15 ponds were surveyed from five locations and all the fish were brought to the laboratory within 4 h of collection in oxygen filled polythene bags. Each live fish was examined thoroughly in the laboratory for the presence of *Argulus *as described by Natarajan [4]. *Argulus *spp. from infected carps were isolated carefully and transferred on to clean grease free glass slides with few drops of distilled water, covered with cover slips and sealed with Distrene, Plasticizer and Xylene (DPX). The slides were examined under 4X lens of upright microscope (Motic BA400) and subsequently under stereomicroscope (Olympus SZ 40). The parasite was identified based on the morphometric criteria [12].

**Determination of parasitic frequency index (PFI) and severity of infection: **The parasitic frequency index (PFI) was calculated by taking the number of hosts infected by *Argulus *against the total number of hosts examined. The severity of infection was determined by following the scale proposed by Lightner [13] with slight modification: 0 = no signs of parasite, 0.5 = a very few scattered signs of parasitic infection; 1 = low parasitic infection; 2 = low to moderate parasitic infection, 3 = moderate parasitic infection, 4 = severe parasitic infection.

**Molecular analysis: **For molecular characterization, two morphologically distinct *Argulus *species, viz., *A. bengalensis *from *L. rohita *(n=5) and *A. siamensis *from *C. carpio *(n=1) were preserved in 70% ethanol and stored at 4ºC until extraction. The DNA was extracted according to protocol described in Anjan *et al. *[14]. The 18S small subunit ribosomal RNA (18S rRNA) was amplified by Gradient PCR system (Eppendorf Master cycler Pro S) using a set of universal eukaryotic primers - ERIB1, 5´-ACC TGG TTG ATC CTG CCA G-3´ and ERIB10, 5´-CTT CCG CAG GTT CAC CTA CGG-3´ [15]. A 25 μL volume of PCR-mixture prepared with 20 mmol/L Tris- HCl (pH=8.4), 50 mmol/L KCl, 2.0 mmol/L MgCl2, 200 umol/L of dNTPs, 1 umol/L of each primer, 30 ng of genomic DNA and 1.5 U of Taq DNA polymerase. Amplification was done by initial denaturation at 95°C for 5 min, followed by 35 cycles of denaturation at 95°C for 30 sec, annealing of primers at 51°C for 30 sec and extension at 72°C for 60 sec. The final extension was at 72°C for 5 min. The PCR amplicons were analysed on a 1.5% agarose gel containing 0.5 μg/mL ethidium bromide in 1X Tris- acetate- EDTA (TAE) buffer. The DNA bands were visualized and documented using a Gel documentation system (Syngene G-Box F3). The amplified PCR product was first purified using EXO-SAP treatment. The concentration of the purified DNA was determined and subjected to automated DNA sequencing on ABI 3730xl Genetic Analyzer (Applied Biosystems, USA). Sequencing was carried out using BigDye® Terminator v3.1 Cycle sequencing kit (Applied Biosystems, USA) following manufacturers’ instructions. Chromatogram of sequence data confirmed the peak, reproducibility, quality of gene sequences. Raw sequence data was arranged as complementary and consensus through online Reverse-Complement tools and Genefisher2. Multiple sequence alignments (MSA) were carried out in 3 states as pair- wise alignments by ClustalW [16]. The nucleotide sequences of 18S rRNA gene of sixteen *Argulus *spp. available in NCBI GenBank database, viz. *A. foliaceus *(EU370428 and JQ740819), *A. nobilis *(M27187), *A. coregoni *(JQ740820), *A. japonicus *(JN558647, KF747855, KF747856, KF747857, KF747858, KF747859, KF747860, KF747861), *A. monodi *(DQ813452), *A. rhipidiophorus *(KF747862) and *Argulus *sp. (DQ531766 and JN558648) were compared with the new 18S rRNA gene sequences of *Argulus *spp. of the present study (Table 2) using the BLAST network service of NCBI (www.ncbi.nlm. gov/BLAST/). The analysis involved 28 nucleotide sequences. Ciliophoran parasite *Ichthyophthirius multifiliis *(U17354) was taken as an out-group. Phylogenetic cluster was based on the alignment of about 1800 bp long sequences. The phylogenetic tree was constructed using the Bayesian inference method implemented in the MrBayes program [v3.2.3] [17]. Four Markov Chain Monte Carlo (MCMC) chains were run for 10,000 generations, sampling every 10 generations. Less than 50% bootstrap replicates were collapsed. The DNA distance matrix (dnadist) was analyzed by Kimura 2- parameter model [18] in MEGA6 [19].

## RESULTS

Screening of 320 fish samples from different geographical areas of West Bengal, India yielded an *Argulus *infection rate of 10.63% (Table 1). Twelve out of 15 surveyed ponds had *Argulus *infection. The parasitic frequency index (PFI) was observed to be 4.65% in *C. catla, *8.09% in *L. rohita, *9.76% in *C. mrigala, *5% in *C. carpio *and 20% each in *H. molitrix *and *L. bata*. The rate of infection was high in September (PFI: 17.02%) and October (PFI: 12.90%). The universal primer sets ERIB1 and ERIB10 successfully amplified approximately 1800 bp fragments of the 18S rRNA gene from *Argulus *spp. The PCR amplified products were sequenced, similarity calculations performed and compared the sequences of approximately 1.8 kb with sequences available in GenBank using BLAST network services and the results are presented in Tables 2 and 3.

**Figure 1:**
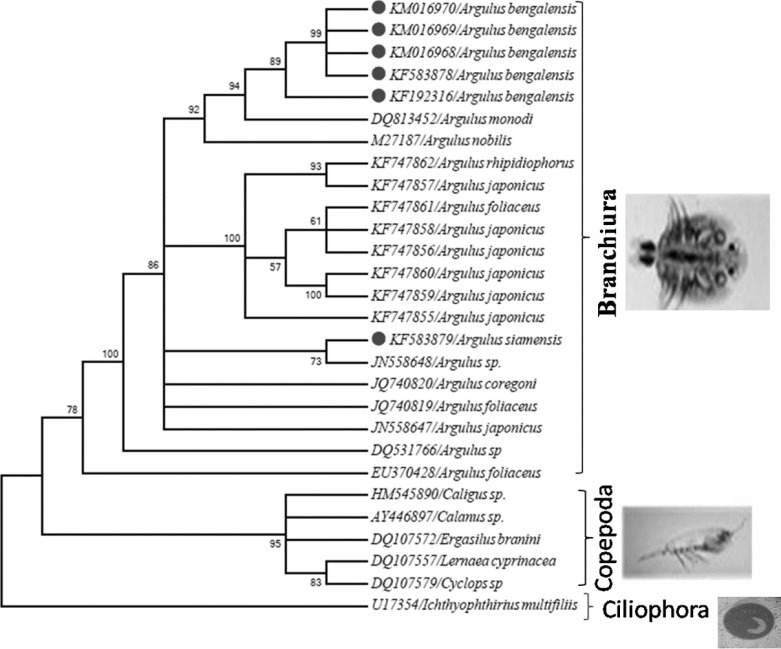
Molecular phylogenetic tree produced by Maximum Likelihood (ML) method on the basis of Bayesian interference theory relationship. GenBank accession number is provided for each species. Scale bar = amount of inferred evolutionary change along the branch lengths

The 18S rRNA sequences of five *A. bengalensis *and one *A. siamensis *of this study were submitted to the GenBank database by using Sequin software under nucleotide accession numbers KF583878*, *KF192316, KM016968, KM016969, KM016970 and KF583879, respectively. In the phylogenetic tree (Fig. 1) all the crustaceans were clustered together as a separate clade with two distinct lineages. In lineage-1, all *Argulus *spp. were clustered together, although with polytomy, giving many temporally based branches.

**Table 1:** Prevalence of *Argulus *infection in cultured carps of West Bengal, India

Sampling period	Number infected	PFI (%)	Severity of infection
June 2012 (n=20)	1	5.00	0.5
September 2012 (n=47)	8	17.02	0.5-1.0
October 2012 (n=124)	16	12.90	0.5
November 2012 (n=25)	2	8.00	0.5
December 2012 (n=41)	2	4.88	0.5
March 2013 (n=63)	5	7.94	0.5-1.0
Fish species			
*Catla catla *(n=43)	2	4.65	0.5
*Labeo rohita *(n=136)	11	8.09	0.5
*Cirrhinus mrigala *(n=41)	4	9.76	0.5-1.0
*Cyprinus carpio *(n=20)	1	5.00	0.5
*Hypophthalmichthys molitrix *(n=20)	4	20.00	1.0
*Labeo bata *(n=60)	12	20.00	0.5
Location			
Haripota (n=67)	9	13.43	0.5-1.0
Bantala (n=124)	16	12.90	0.5
Garia and Chakgaria (n=104)	7	6.73	0.5-1.0
Memari (n=25)	2	8.00	0.5

Values in parenthesis are the numbers of fish examined

**Table 2:** Identification and molecular characterization of *Argulus *spp. of carps

*Argulus *species	Host species	Farm	Size of	edited	Accession
*Argulus bengalensis*	*Labeo rohita*	Bongaon	1680		KM016968
*Argulus bengalensis*	*Labeo rohita*	Naihati	1664		KM016969
*Argulus bengalensis*	*Labeo rohita*	Naihati	1652		KM016970
*Argulus bengalensis*	*Labeo rohita*	Haripota	1699		KF192316
*Argulus bengalensis*	*Labeo rohita*	Chakgaria	1823		KF583878
*Argulus siamensis*	*Cyprinus carpio*	Chakgaria	1714		KF583879

The nucleotide sequences revealed 77-99% homology among the 18S rRNA gene sequences of other related species available in NCBI Genbank database. The sequence homogensity of the presently described Indian *Argulus *spp. with sixteen other *Argulus *spp. described from outside India was 87–99%. Evolutionary pair-wise distances between *A. bengalensis *(KF583878, KF192316, KM016968, KM016969 and KM016970) and other *Argulus *spp. measured by Kimura-2 parameter algorithm ranged from 0 to 14.80%. Likewise, it ranged from 0 (*Argulus *sp. JN558648) to 13.30% (*A. japonicus *KF747855 and KF747856) for *A. siamensis *KF583879. Evolutionary pair- wise distances between *A. bengalensis *and other analysed species (Table 4) varied from 5.90 (*Caligus *sp. HM545890) to 19.80% (*Ichthyophthirius multifiliis *U17354). In case of *A. siamensis *the evolutionary pair-wise distances with other species varied from 5.90 (*Ergasilus branini *DQ107572) to 20.20% (*Ichthyophthirius multifiliis *U17354). In lineage-2, other tested crustaceans gene sequences from NCBI GenBank database were clustered together.

**Table 3:** Homogeneity of 18S rRNA gene sequences of *Argulus *spp. and related species available in GenBank database

***Argulus *** **spp. and related**	**NCBI** **Accession**	**Homogeneity (%)**					
**group from** **NCBI database**	**number**	**KF583878**	**KF583879**	**KF192316**	**KM016968**	**KM016969**	**KM016970**
*Argulus rhipidiophorus* *A. foliaceus*	KF747862 KF747861	91 91	97 97	96 96	91 91	89 90	89 89
*A. japonicus*	KF747860	89	66	93	89	88	87
*A. japonicus*	KF747859	89	96	92	89	88	87
*A. japonicus*	KF747858	91	97	96	91	89	89
*A. japonicus*	KF747857	91	98	96	91	90	89
*A. japonicus*	KF747856	91	97	96	91	89	89
*A. japonicus*	KF747855	91	98	96	91	90	89
*A. japonicus*	JQ740820	92	99	97	92	91	90
*A. coregoni*	JQ740819	92	99	97	92	91	90
*A.foliaceus*	JN558648	92	99	97	92	91	90
*Argulus *sp.	JN558647	92	98	97	92	91	90
*A. japonicus*	DQ813452	92	96	97	92	90	91
*A. monodi*	EU370428	90	82	82	89	88	82
*A. foliaceus*	DQ531766	93	95	96	93	92	88
*Argulus *sp.	M27187	84	97	95	84	83	91
*A. nobilis*	HM545890	84	85	88	84	83	83
*Caligus *sp.	AY446897	83	89	89	83	82	85
*Calanus *sp*.*	DQ107572	82	85	87	82	81	83
*Ergasilus branini Lernaea*	DQ107557 DQ107579	79 85	84 83	87 86	85 82	85 82	82 81
*cyprinacea Cyclops *sp.	U17354	80	78	77	80	80	80
*Ichthyophthirius multifiliis *Present study *A. bengalensis*	KF583878	100	92	94	100	99	98
*A. siamensis*	KF583879	92	100	91	92	91	91
*A. bengalensis*	KF192316	91	94	100	91	90	90
*A. bengalensis*	KM016968	100	92	91	100	99	98
*A. bengalensis*	KM016969	99	91	90	99	100	99
*A. bengalensis*	KM016970	98	91	90	98	99	100

## DISCUSSION

Members of the genus *Argulus *spent major part of the life in surface and column waters and may have some predilection towards surface feeder, but enjoys both surface and column niches. Although 12 out of 15 surveyed ponds (80%) had *Argulus *infection, the infection rate of 10.63% in fish indicated a low prevalence. Contrarily, Paria and Konar [20] reported that 0.8-9.8% the ponds were affected out of the total 1332 freshwater fish ponds surveyed. According to them, *Argulus *was a not significant problem in West Bengal. The PFI was observed to be high in *H. molitrix *and *L. bata *followed by *L. rohita, C. mrigala, C. carpio *and *C. catla*. Contrary to this study, *Argulus *infection was reportedly more in *Catla catla *[21]; while Shella *et al*. [22]observed that *L. rohita *was susceptible to argulosis. The present study recorded high rate of *Argulus *infection in September (PFI: 17.02%) and October (PFI: 12.90%), i.e., the late rainy season. Earlier studies [23, 24], however, recorded the highest *Argulus *infestation in winter and least in rainy season; while other studies [25, 26] recorded incidence of argulosis throughout the year. Further, the fish from Haripota (PFI: 13.43%) and Bantala (PFI: 12.90%) had high *Argulus *infection than other locations, probably due to the presence of high organic loads, as the ponds from these localities use sewage water to raise fish. The infected fish exhibited a very few scattered signs of parasitic infection to low parasitic infection.

**Table 4:** Estimates of evolutionary divergence (in %) between sequences of *Argulus *spp. and out group species available in GenBank

	**Crustacean species**	**NCBI**	**1**	**2**	**3**	**4**	**5**	**6**	**7**	**8**	**9**	**10**	**11**
1	*Argulus bengalensis*	KM016970											
2	*A. bengalensis*	KM016969	1.00										
3	*A. bengalensis*	KM016968	1.00	0.00									
4	*A. bengalensis*	KF583878	1.00	0.00	0.00								
5	*A. bengalensis*	KF192316	1.30	3.00	3.00	3.00							
6	*A. siamensis*	KF583879	2.10	1.60	1.60	1.60	1.30						
7	*A.rhipidiophorus*	KF747862	9.90	9.30	9.30	9.30	9.60	8.50					
8	*A. foliaceus*	KF747861	9.90	9.30	9.30	9.30	9.60	8.50	0.00				
9	*A. japonicus*	KF747860	14.80	14.20	14.20	14.20	14.50	13.30	4.30	4.30			
10	*A. japonicus*	KF747859	14.80	14.20	14.20	14.20	14.50	13.30	4.30	4.30	0.00		
11	*A. japonicus*	KF747858	9.90	9.30	9.30	9.30	9.60	8.50	0.00	0.00	4.30	4.30	
12	*A. japonicus*	KF747857	9.90	9.30	9.30	9.30	9.60	8.50	0.00	0.00	4.30	4.30	0.00
13	*A. japonicus*	KF747856	9.90	9.30	9.30	9.30	9.60	8.50	0.00	0.00	4.30	4.30	0.00
14	*A. japonicus*	KF747855	9.90	9.30	9.30	9.30	9.60	8.50	0.00	0.00	4.30	4.30	0.00
15	*A. coregoni*	JQ740820	1.80	1.30	1.30	1.30	1.60	3.00	8.20	8.20	13.00	13.00	8.20
16	*A. foliaceus*	JQ740819	1.80	1.30	1.30	1.30	1.60	3.00	8.20	8.20	13.00	13.00	8.20
17	*Argulus *sp.	JN558648	2.10	1.60	1.60	1.60	1.30	0.00	8.50	8.50	13.30	13.30	8.50
18	*A. japonicus*	JN558647	1.80	1.30	1.30	1.30	1.60	3.00	8.20	8.20	13.00	13.00	8.20
19	*A. monody*	DQ813452	1.60	1.00	1.00	1.00	1.00	1.30	9.30	9.30	14.20	14.20	9.30
20	*Argulus *sp.	DQ531766	2.90	2.40	2.40	2.40	2.60	1.30	9.30	9.30	13.90	13.90	9.30
21	*A. foliaceus*	EU370428	12.80	12.50	12.50	12.50	12.20	12.10	19.80	19.80	25.20	25.20	19.80
22	*A. nobilis*	M27187	1.80	1.30	1.30	1.30	1.60	3.00	8.20	8.20	13.00	13.00	8.20
23	*Caligus *sp.	HM545890	6.50	5.90	5.90	5.90	5.90	5.90	14.50	14.50	19.90	19.90	14.50
24	*Calanus *sp.	AY446897	8.20	8.60	8.60	8.60	8.30	8.20	16.90	16.90	22.10	22.10	16.90
25	*Ergasilus branini*	DQ107572	6.80	6.50	6.50	6.50	6.50	5.90	14.20	14.20	19.50	19.50	14.20
27	*Lernaea cyprinacea*	DQ107557	7.10	6.50	6.50	6.50	6.50	6.20	14.50	14.50	19.50	19.50	14.50
27	*Cyclops *sp.	DQ107579	10.60	10.30	10.30	10.30	10.30	10.30	18.80	18.80	24.10	24.10	18.80
28	*Ichthyophthirius multifiliis*	U17354	19.80	19.50	19.50	19.50	19.50	2.20	30.00	30.00	35.90	35.90	30.00

The objective of the present study was to characterize Indian *Argulus *spp. infecting the carps cultured in West Bengal, India by molecular tools as the 18S rDNA gene is the most conserved in nature and provide valuable information for comparative analysis [27]. In our study, the universal primer sets ERIB1 and ERIB10 successfully amplified approximately 1800 bp fragments of the 18S rRNA gene from *Argulus *spp. Earlier, there have been reports on the partial sequence of 28S rRNA of *Argulus *sp. [8], comparative phylogenetic analysis of Pentastomida and complete mitochondrial DNA (mt DNA) of *A. americanus *[10], molecular phylogeny of Branchiura, Pentastomida and other Maxillopoda based on mitochondrial 16S rRNA, nuclear 18S and 28S rRNA [28] and genetic variability within mtDNA regions of fish louse *A. japonicus *from Africa, Middle East and Asia [9]. These genetic data and phylo-geographical structure of a species provide the basic information required to understand their evolution and biogeographical history. According to Alam and Khan [29] populations having higher similarity are more homogeneous groups. The NCBI sequence similarity matrix between query and reference sequences of *Argulus *spp. showed genetic heterogeneity because the isolates were collected from different fish species and locations. This was also reflected in the phylogenetic tree (Fig. 1). Although all *Argulus *spp. were clustered together, polytomy was noticed possibly due to genetic heterogeneity. Similarly, Wadeh *et al*. [9] reported the genetic variability within fish louse *A. japonicus *from Africa, Middle East and Asia by examining sequence variability between cytochrome C oxidase subunit 1 (cox1) and NADH dehydrogenase subunits 1 and 4 (nad1 and nad4).

In a similar study, Sahoo *et al*. [11] reported the genetic variation of *Argulus *species collected from freshwater aquaculture systems of India by RAPD analysis. Their results indicated that the RAPD banding patterns obtained using host DNA were clearly distinct from that of parasites of all locations. They also constructed phylogenetic tree of *Argulus *species of India, but based on Nei’s genetic distance and, hence, it is difficult to compare with their study. Both studies, however, recorded genetic heterogeneity among *Argulus *spp. collected from different fish species and locations. Yet, this is the first record of 18S rRNA gene sequence based molecular characterization of *A. bengalensis *and *A. siamensis *from India and their phylogenetic relationship with other *Argulus *spp. available in GenBank database. The molecular method appears to be a reliable diagnostic tool for *Argulus *characterization and can be useful for identifying *Argulus *spp. Our attempt to estimate genetic diversity among Indian *Argulus *spp., provided the baseline data for future work on population structure analysis of *Argulus *species, as very limited information exist for this economically important species, which would help identifying and managing these parasites in aquaculture.
